# Smoking and drinking habits relating to development of ulcerative colitis in Japanese: A multicenter case–control study

**DOI:** 10.1002/jgh3.12857

**Published:** 2022-12-29

**Authors:** Kyoko Kondo, Yu Ono, Satoko Ohfuji, Kenji Watanabe, Hirokazu Yamagami, Mamoru Watanabe, Yuji Nishiwaki, Wakaba Fukushima, Yoshio Hirota, Yasuo Suzuki, for the Japanese Case‐Control Study Group for Ulcerative Colitis

**Affiliations:** ^1^ Management Bureau Osaka Metropolitan University Hospital Osaka Japan; ^2^ Department of Public Health Osaka Metropolitan University Graduate School of Medicine Osaka Japan; ^3^ Center for Inflammatory Bowel Disease, Division of Internal Medicine Hyogo College of Medicine Hyogo Japan; ^4^ Department of Gastroenterology Osaka Metropolitan University Graduate School of Medicine Osaka Japan; ^5^ Department of Gastroenterology and Hepatology, School of Medicine Tokyo Medical and Dental University Tokyo Japan; ^6^ Department of Environmental and Occupational Health, School of Medicine Toho University Tokyo Japan; ^7^ Clinical Epidemiology Research Center SOUSEIKAI Medical Group (Medical Co. LTA) Fukuoka Japan; ^8^ Inflammatory Bowel Disease Center Toho University Sakura Medical Center Chiba Japan

**Keywords:** case–control study, drinking habits, smoking habits, ulcerative colitis

## Abstract

**Background and Aim:**

The number of patients with ulcerative colitis (UC) has been increasing in Japan. To elucidate the risk factors for developing UC in Japan, a hospital‐based case–control study was conducted. This study examined the association between smoking/drinking habits and UC onset in detail.

**Methods:**

Cases comprised 132 Japanese patients who had been newly diagnosed with UC between 2008 and 2014 at 38 collaborating hospitals in Japan, and controls comprised 167 patients without UC. Detailed data on smoking and drinking habits were collected using a self‐administered questionnaire.

**Results:**

Ex‐smokers showed an increasing odds ratio (OR) for UC development compared with never smokers (OR 2.42, 95% confidence interval 1.24–4.72). The ORs of ex‐smokers were particularly high among subjects aged less than 40 years, subjects who had smoked more than 10 pack‐years, and subjects who were within 13 years of quitting smoking. Regarding drinking habits, ex‐drinkers also showed a more than twofold higher OR for UC compared to never drinkers. Ex‐drinkers 40 years or older, ex‐drinkers who had consumed more than 364 drink‐years, and subjects who were less than 6 years after quitting drinking showed increased ORs for UC.

**Conclusion:**

These findings suggest the need for careful attention for UC onset among heavy smokers who quit smoking before 40 years of age and heavy drinkers who quit drinking at ≥40 years of age.

## Graphical abstract

The present multicenter case‐control study using incident (new) cases of UC showed a higher OR for UC among ex‐smokers and ex‐drinkers. Our findings suggest the need for careful attention for UC onset in heavy smokers who quit smoking before 40 years old and in heavy drinkers who quit drinking at or after 40 years old.

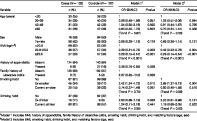

## Introduction

The incidence of ulcerative colitis (UC) has been showing an increasing trend in many parts of the world. Even in Japan, which was previously thought to have a low incidence rate, UC has been increasing over the past several decades.^1^, ^2^, ^3^, ^4^ Many genetic predispositions and environmental factors, such as appendectomy and cigarette smoking, have been suggested as factors affecting the development of UC.^5^, ^6^ Although the mechanisms underlying the effects of these environmental factors on the onset of UC remain largely unknown, associations between cigarette smoking status and UC development have been investigated in several studies.^7^, ^8^ Interestingly, the most consistent findings show that current smokers have reduced risk for UC whereas ex‐smokers have increased risk.^9^, ^10^, ^11^, ^12^, ^13^ However, with respect to ex‐smokers, most associations have been reported from case–control studies in which prevalent cases (individuals previously diagnosed with UC) were used instead of incident cases (newly diagnosed cases). As a result, ex‐smokers who served as cases in those studies might actually have been smokers at the time of UC onset because of reasons such as patients quitting smoking due to gastrointestinal symptoms caused by UC. Thus, apparent risks could potentially have been detected as a result of reverse causality. In addition, if quitting smoking is confirmed as an actual risk factor for UC, identifying groups at higher risk of developing UC after quitting smoking will be important, since smoking cessation is being heavily promoted around the world to prevent various diseases.

On the other hand, the effects of drinking on the risk of UC have been examined in a small number of prior studies,^11^, ^14^, ^15^ but the association remains controversial.

The aim of this study was therefore to explore the details of the association between smoking/drinking habits and UC in a multicenter, collaborative, hospital‐based, case–control study in Japan. In this case–control study, we minimized the problem of reverse causality from patients quitting smoking and/or drinking due to the onset of UC symptoms by prospectively registering newly diagnosed UC cases. In addition, using detailed data, we investigated which groups are strongly affected by smoking/drinking cessation in terms of the development of UC.

## Methods

### Subjects

The methods used for the present study have been described elsewhere.^16^, ^17^, ^18^ Briefly, from September 2008 to March 2014, a multicenter case–control study was conducted with the cooperation of 38 facilities in Japan.

Cases were prospectively registered among Japanese patients under 80 years of age who were newly diagnosed with UC according to the diagnostic criteria proposed by the Research Committee of Inflammatory Bowel Disease in 2008. Patients referred from other hospitals were allowed to register if they had been diagnosed with UC within the preceding 3 months.

Regarding controls, as far as possible, two patients with matching sex and age within the same 5‐year age category were recruited for each case, from the same hospital as the case. We asked for researchers to select one control from the gastroenterology department and the other one from another department such as orthopedics, ophthalmology, or a general medical department. Exclusion criteria were the presence of malignancy, persistent symptoms of diarrhea and abdominal pain for more than 1 week, or a history of inflammatory bowel disease (IBD).

### Collection of information

For cases, the following clinical information was collected by physicians using a standardized questionnaire: date of registration to this study, date of first visit to the hospital, date of symptom onset, date of diagnosis, disease severity at diagnosis (mild, moderate, severe, or fulminant), and disease site at diagnosis (rectum, colon, cecum, or ileum). In addition, the following information was obtained from a self‐administered questionnaire: demographic characteristics, height, weight, history of appendicitis, family history of UC, smoking habit, and drinking habit. Information on smoking habit was collected in the following three categories: non‐smoker, ex‐smoker, or current smoker. For ex‐smokers, the age at quitting smoking and the reasons for quitting were also asked. Furthermore, for ex‐smokers and current smokers, the age at which smoking started and the average number of cigarettes smoked daily were investigated. Information on drinking habits was collected in the following three categories: non‐drinker, ex‐drinker, or current drinker. For ex‐drinkers, the age at quitting drinking and the reasons for quitting were also investigated. Furthermore, for ex‐drinkers and current drinkers, the age at which drinking started and drinking habits during the “peak” period of highest alcohol consumption were investigated. We assessed drinking frequency, period (age at starting, age at stopping), and volume of alcohol intake according to beverage types.

### Analysis

Because this was a hospital‐based case–control study, subjects might have quit smoking or drinking because they developed an illness. Subjects who had quit smoking or drinking within 1.5 years before study entry were thus defined as current smokers or current drinkers. Family history of UC was examined in relatives up to the second degree.

The cumulative amount of cigarette smoking was calculated using the following equation: pack‐years of cigarettes smoking (pack‐years) = average number of cigarettes smoked daily/20 (assuming 20 cigarettes per pack) × duration of smoking (years). The cumulative amount of alcohol consumption during the “peak” period was calculated using the following equation: cumulative alcohol consumption (drink‐years) = amount of ethanol consumed each week (g) × duration of “peak” period in which alcohol consumption was highest (years). These variables were classified where possible as quartiles (or dichotomies) according to the distribution of control subjects.

Logistic regression model was used to calculate the odds ratio (OR) and 95% confidence interval (95%CI) of each variable for UC development. Trends for association were assessed by assigning ordinal scores to each category. In multivariate analysis, the model included variables that showed significantly different distributions when comparing cases and controls and variables suggested as risk factors for UC from previous studies.

Sub‐analysis classified subjects into two or three groups according to sex (male/female), age (<40 years/≥40 years), severity (mild/moderate or severe), and disease site (rectum/colon/appendix or ileum). Non‐smokers or non‐drinkers were used as references to investigate the associations between smoking or drinking habits and UC.

Next, the effects of past smoking on the development of UC among ex‐smokers and the effects of past drinking on the development of UC among ex‐drinkers were analyzed in detail. Subjects of analysis were non‐smokers and ex‐smokers, and non‐drinkers and ex‐drinkers, respectively, excluding current smokers and current drinkers.

The level of significance was set at 0.05. Statistical analyses were conducted using SAS version 9.3 (SAS Institute Inc., Cary, NC).

### Ethical considerations

The study protocol was approved by the ethics committees at Osaka City University Graduate School of Medicine and all other collaborating hospitals, and the study was performed in accordance with the Declaration of Helsinki. Written informed consent was obtained from all subjects prior to enrolment. In the case of subjects under 20 years of age, written informed consent was obtained from their legal representatives.

## Results

Of the 151 cases and 199 controls who met the inclusion criteria, responses to the questionnaire were obtained from 133 cases and 167 controls (response rates: 88% and 84%, respectively). Of these, one case who had not described his drinking habit in the self‐administered questionnaire was excluded. As a result, 132 cases and 167 controls were included in the final analysis. In terms of matching conditions for these 299 participants, 1:2 matching was achieved in 43 pairs (43 cases, 86 controls) and 1:1 matching in 43 pairs (43 cases, 43 controls). The remaining 46 cases and 38 controls were unmatched. When conditional logistic modeling was used, the number of subjects for analysis was reduced to 215. As a result, 299 subjects (132 cases, 167 controls) were analyzed using an unconditional logistic model, which included matching factors (age and sex).

Controls were selected from gastroenterology departments and other departments in approximately 1:1 ratio. The other departments were most frequently orthopedic surgery, followed by general medicine, nephrology, ophthalmology, rheumatology, and others.

Table 1 shows disease characteristics for the cases. Median age at symptom onset was 41.4 years. Median time from symptom onset to enrollment was 2.4 months, and nearly 90% of cases were enrolled within 1 year after symptom onset. As for disease severity at diagnosis, 16% of cases were severe. The site of UC at the time of diagnosis was rectal in 21% and appendix/ileum in about 30%.

**Table 1:** Disease characteristics of cases (*N* = 132)

		*n*	(%)
Age at symptom onset (years)	Median (range)	41.4	(8.7–74.8)
	<30	23	(24)
	30–39	22	(23)
	40–49	24	(26)
	≥50	25	(27)
	Missing	38	
Time from onset to enrollment (months)	Median (range)	2.4	(0–172.8)
	<4	63	(67)
	4–11	21	(22)
	12–17	4	(4)
	≥18	6	(6)
	Unknown	38	
Time from initial examination to enrollment (months)	Median (range)	1.2	(0–36.0)
	<4	110	(86)
	4–11	14	(11)
	≥12	4	(3)
	Unknown	4	
Severity	Mild	40	(41)
	Moderate	41	(42)
	Severe	16	(16)
	Fulminant	0	(0)
	Unknown	35	
Disease site	Rectum	21	(21)
	Colon	44	(45)
	Appendix	30	(31)
	Ileum	3	(3)
	Unknown	34	

Table 2 shows the factors associated with the development of UC (main analysis). First, we conducted a multivariate analysis (Model 1) in which the model included body mass index (BMI), history of appendicitis, family history of UC, smoking habits, drinking habits, and matching factors (age, sex). Compared to non‐smokers, ex‐smokers showed an increased OR (OR 2.42, 95% CI 1.24–4.72, *P* = 0.010). OR appeared to be increased in ex‐drinkers compared to non‐drinkers (OR 2.53, 95% CI 0.89–7.19, *P* = 0.083). Model 1 may have been unstable because of the small number of individuals with a “present” history of appendicitis and a “present” family history of UC. When we examined Model 2 excluding these two variables, the results were almost the same as those for Model 1, so Model 2 was adopted for subsequent analyses.

**Table 2:** Factors associated with the development of ulcerative colitis (main analysis)

		Cases (*N* = 132)	Controls (*N* = 167)	Model 1^†^		Model 2^‡^	
Variable		*n* (%)	*n* (%)	OR (95%CI)	*P*‐value	OR (95%CI)	*P‐*value
Age (years)	<30	33 (25)	38 (23)	1		1	
	30–39	34 (26)	40 (24)	0.98 (0.48–1.99)	0.957	1.03 (0.51–2.06)	0.944
	40–49	31 (23)	42 (25)	1.04 (0.50–2.16)	0.920	0.91 (0.44–1.87)	0.795
	≥50	34 (26)	47 (28)	0.83 (0.40–1.73)	0.622	0.70 (0.34–1.44)	0.335
				(Trend *P* = 0.67)		(Trend *P* = 0.30)	
Sex	Male	76 (58)	84 (50)	1		1	
	Female	56 (42)	83 (50)	0.68 (0.39–1.19)	0.174	0.66 (0.38–1.14)	0.131
BMI (kg/m^2^)	<20.8	69 (52)	53 (32)	1		1	
	20.8–23.5	36 (27)	57 (34)	0.39 (0.21–0.72)	0.003	0.42 (0.23–0.76)	0.004
	≥23.6	27 (20)	57 (34)	0.29 (0.15–0.57)	<0.001	0.28 (0.14–0.54)	<0.001
				(Trend *P* < 0.001)		(Trend *P* < 0.001)	
History of appendicitis	Absent	124 (94)	140 (84)	1			
	Present	8 (6)	27 (16)	0.38 (0.16–0.95)	0.038		
Family history of ulcerative colitis	Absent	123 (93)	162 (97)	1			
	Present	9 (7)	5 (3)	2.87 (0.82–10.0)	0.099		
Smoking habit	No	67 (51)	99 (59)	1		1	
	Ex‐smoker	45 (34)	29 (17)	2.42 (1.24–4.72)	0.010	2.65 (1.37–5.13)	0.004
	Current smoker	20 (15)	39 (23)	0.74 (0.37–1.48)	0.397	0.80 (0.40–1.58)	0.516
				(Trend *P* = 0.70)		(Trend *P* = 0.88)	
Drinking habit	No	37 (28)	62 (37)	1		1	
	Ex‐drinker	15 (11)	9 (5)	2.53 (0.89–7.19)	0.083	2.20 (0.81–5.98)	0.121
	Current drinker	80 (61)	96 (57)	1.24 (0.71–2.16)	0.447	1.19 (0.69–2.05)	0.532
				(Trend *P* = 0.55)		(Trend *P* = 0.63)	

^†^ Model 1 includes BMI, history of appendicitis, family history of ulcerative colitis, smoking habit, drinking habit, and matching factors (age, sex).

^‡^ Model 2 includes BMI, smoking habit, drinking habit, and matching factors (age, sex).

We created subgroups for each sex, age, severity, and disease site, and examined the association between smoking/drinking habits and development of UC (Table 3, subgroup analysis). Variables included in the model were the same as in Model 2 of the main analysis. For ex‐smokers compared with non‐smokers, males in the sex subgroup (OR 2.84, 95% CI 1.17–6.90), younger than 40 years in the age subgroup (OR 9.57, 95% CI 2.67–34.3), moderate or severe in the severity subgroup (OR 4.83, 95% CI 2.02–11.6), and appendix or ileum in the disease site subgroup (OR 7.26, 95% CI 2.46–21.4) showed increased ORs for the development of UC. When comparing current smokers and non‐smokers, no subgroups were associated with the development of UC.

**Table 3:** Association between smoking and drinking habits and development of ulcerative colitis (subgroup analyses)

		Smoking habit			Drinking habit		
		Adjusted OR (95%CI) (ref. Non‐smoker)			Adjusted OR (95%CI) (ref. Non‐drinker)		
		Ex‐smoker	Current smoker	*P* for trend	Ex‐drinker	Current drinker	*P* for trend
All participants^†^	(132 cases/167 controls)	2.65 (1.37–5.13)	0.80 (0.40–1.58)	0.88	2.20 (0.81–5.98)	1.19 (0.69–2.05)	0.63
Sex^‡^							
Male	(76 cases/84 controls)	2.84 (1.17–6.90)	1.10 (0.44–2.73)	0.84	2.36 (0.54–10.3)	1.41 (0.64–3.15)	0.50
Female	(56 cases/83 controls)	2.67 (0.84–8.54)	0.39 (0.11–1.34)	0.41	1.98 (0.47–8.30)	1.03 (0.47–2.24)	0.96
Age (years)							
<40^§^	(67 cases/78 controls)	9.57 (2.67–34.3)	0.89 (0.33–2.37)	0.60	0.77 (0.13–4.48)	0.87 (0.40–1.93)	0.75
≥40^¶^	(65 cases/89 controls)	1.23 (0.51–2.96)	0.58 (0.21–1.60)	0.34	5.26 (1.32–21.0)	1.50 (0.68–3.28)	0.46
Severity^†^							
Mild	(40 cases/167 controls)	1.55 (0.59–4.07)	0.42 (0.12–1.44)	0.26	3.48 (0.71–17.1)	2.13 (0.84–5.40)	0.15
Moderate/severe	(57 cases/167 controls)	4.83 (2.02–11.6)	1.16 (0.46–2.96)	0.38	3.02 (0.91–10.1)	0.84 (0.40–1.78)	0.53
Disease site^†^							
Rectum	(21 cases/167 controls)	1.43 (0.42–4.84)	0.35 (0.07–1.77)	0.27	1.82 (0.17–19.4)	2.38 (0.71–8.06)	0.16
Colon	(44 cases/167 controls)	2.36 (0.93–6.01)	0.55 (0.17–1.82)	0.64	1.86 (0.43–8.12)	0.91 (0.41–2.03)	0.77
Appendix/ileum	(33 cases/167 controls)	7.26 (2.46–21.4)	1.98 (0.64–6.10)	0.14	5.42 (1.38–21.3)	0.98 (0.37–2.62)	0.72

^†^ Adjusted for BMI, smoking habit, drinking habit, and matching factors (age, sex).

^‡^ Adjusted for BMI, smoking habit, drinking habit, and matching factors (age).

^§^ Adjusted for BMI, smoking habit, drinking habit, and matching factors (age: <30 or 30–39, sex).

^¶^ Adjusted for BMI, smoking habit, drinking habit, and matching factors (age: 40–49 or ≥50, sex).

When comparing ex‐drinkers and non‐drinkers, 40 years and older in the age subgroup (OR 5.26, 95% CI 1.32–21.0) and appendix or ileum in the disease site subgroup (OR 5.42, 95% CI 1.38–21.3) showed increased ORs for the development of UC. When comparing current drinkers with non‐drinkers, no subgroup was associated with development of UC.

Next, we examined association between ex‐smoking and ex‐drinking and the development of UC (Table 4). The variables included in this model were the same as in Model 2 of the main analysis. In ex‐smokers, the OR for the development of UC increased with increasing cumulative number of cigarettes smoked (trend *P* = 0.001) and increased significantly beyond 10 pack‐years. OR for the time since quitting was significantly higher for 1–5 years (OR 6.46, 95% CI 2.37–17.6) and 5–13 years (OR 3.26, 95% CI 1.18–9.00), but it did not increase for ≥13 years.

**Table 4:** Association between ex‐smoking and ex‐drinking and development of ulcerative colitis

		Cases	Controls		
		*n* (%)	*n* (%)	OR^†^ (95%CI)	*P*‐value
Cumulative cigarette smoking (pack‐years)					
Non‐smoker		67 (60)	99 (77)	1.00	
Ex‐smoker	<4.0	11 (10)	7 (5)	2.51 (0.87–7.27)	0.091
	4.0–9.9	7 (6)	7 (5)	1.83 (0.56–5.96)	0.319
	10.0–21.0	10 (9)	7 (5)	3.17 (1.00–10.0)	0.049
	≥21.1	17 (15)	8 (6)	5.25 (1.70–16.3)	0.004
				(Trend *P* = 0.001)	
Time since quitting (years)					
Non‐smoker		67 (60)	99 (77)	1.00	
Ex‐smoker	1.5–5.0	23 (21)	7 (5)	6.46 (2.37–17.6)	<0.001
	5.1–13.0	16 (14)	8 (6)	3.26 (1.18–9.00)	0.023
	13.1–22.0	2 (2)	7 (5)	0.52 (0.09–2.84)	0.447
	>22	4 (4)	7 (5)	0.97 (0.23–4.09)	0.970
				(Trend *P* = 0.389)	
Cumulative alcohol consumption (drink‐years)					
Non‐drinker		37 (71)	62 (89)	1.00	
Ex‐drinker	<364	3 (6)	4 (6)	0.97 (0.16–5.97)	0.973
	≥364	12 (23)	4 (6)	5.27 (0.97–28.5)	0.054
	Unknown		1		
				(Trend *P* = 0.075)	
Time since quitting (years)					
Non‐drinker		37 (71)	62 (87)	1.00	
Ex‐drinker	1.5–<6	11 (21)	5 (7)	3.85 (0.88–16.9)	0.074
	≥6	4 (8)	4 (6)	1.01 (0.17–6.15)	0.989
				(Trend *P* = 0.387)	

^†^ Adjusted for BMI, smoking habit or drinking habit, and matching factors (age, sex).

An increased OR for the development of UC was suggested in ex‐drinkers who consumed a large amount of alcohol (≥364 drink‐years in cumulative alcoholic consumption) (OR 5.27, 95% CI 0.97–28.5). In terms of the time since quitting drinking, OR was marginally increased for less than 6 years (OR 3.85, 95% CI 0.88–16.9) but approached 1 for 6 years or more.

## Discussion

An association between the development of UC and ex‐smoking/ex‐drinking was suggested in the present analysis. Subgroup analysis showed the effects of previous smoking to be stronger in men or those younger than 40 years and the effects of previous drinking to be stronger in those 40 years or older. The effects of smoking and drinking cessation were more pronounced in participants with more severe or more wide‐ranging disease, indicating that cessation may also impact the severity and extent of UC. A detailed investigation of smoking cessation showed that the ORs for ex‐smokers were particularly high among heavy smokers and those who had quit within the past 5 years. A detailed investigation of the cessation of alcohol consumption showed that the ORs for ex‐drinkers were particularly high among heavy drinkers and those who had quit within the past 5 years. The association between ex‐drinkers and UC onset represents particularly an important result that has not been reported previously.

The higher OR identified in ex‐smokers agrees with findings from previous studies.^9^, ^10^, ^11^, ^12^, ^13^ Since the present study included only incident (new) cases of UC, higher OR in ex‐smokers was unlikely to have resulted from reverse causality. The OR of participants with a short period after smoking cessation was increased, and the longer the period since smoking cessation, the closer the OR was to that of non‐smokers. These findings suggest that smoking cessation is associated with UC onset, as reported previously. The mechanism may involve nicotine decreasing blood flow during smoking, inhibiting inflammatory mediators from reaching the rectal mucosal surface, and suppressing inflammatory responses, with subsequent smoking cessation transiently increasing the immune function.^10^ Therefore, when a person with a large cumulative load of cigarettes smoked quits smoking at <40 years of age, careful follow‐up may be required for around 5 years after quitting. Given the results of previous studies, use of nicotine patches may be beneficial in the treatment of UC when quitting smoking.^19^, ^20^ Recent reviews have shown that transdermal nicotine, in combination with conventional treatments, is more beneficial in the treatment of UC than conventional treatments alone, but further studies are needed to determine the appropriate doses.^21^ On the other hand, a recent study based on smoking status as of 2 years before the diagnosis of UC showed that smoking cessation was not associated with a worse course of illness.^22^ The present analysis did not find a lower OR in current smokers.

In this study, the OR of ex‐drinkers ≥40 years was increased. A few studies have examined the association between drinking habits and development of UC, but the conclusions were inconsistent.^11^, ^14^, ^15^, ^23^ Since the present study included only incident (new) cases of UC, the higher OR in ex‐drinkers was unlikely to have resulted from reverse causality. The ORs of individuals with a large cumulative alcohol intake or with a short period of abstinence were increased. ORs of those with a small amount of cumulative drinking or a long period of abstinence decreased to the same level as never drinkers. In the future, detailed examinations of the underlying mechanisms will be required, but when an individual with a large cumulative alcohol intake abstains from alcohol above the age of 40 years, follow‐up will be required for around 6 years after quitting drinking.

In our study, in the case of BMI, which we used as an adjusted factor, we identified an association between low BMI and development of UC. BMI information was taken at the time of enrollment, and 89% of cases were enrolled within 1 year of onset, and no association was apparent between BMI and severity or disease site. However, a prospective European cohort study found no association between BMI and development of UC.^24^ In a recent systematic review, both underweight and obese status were independently associated with Crohn's disease, but BMI was not significantly associated with UC.^25^

The present study has three main strengths. First, the possibility of reverse causality was minimized by including only patients with newly diagnosed UC. Second, rigorous enrollment criteria were established to ensure that only patients with newly diagnosed UC were included. As detailed in previous studies,^16^, ^17^, ^18^ researchers in gastroenterology in this study reached definitive diagnoses according to the diagnostic criteria of this research group, resulting in high diagnostic reliability. Third, the careful study design enabled the use of detailed questions, resulting in the collection of detailed information. Cohort studies of low‐incidence diseases such as UC require very large cohort sizes to ensure outcome occurrence that will allow valid statistical analysis. Time as well as financial and human resource constraints generally make such studies unfeasible. In this study, we could obtain detailed information by using a case–control study design with the cooperation of study subjects and the gastroenterologist in charge.

The present study has four main limitations. The first involves recall bias. The accuracy of the self‐reported information collected for smoking and drinking histories may differ according to how well the participants remembered this information. The degree of recall of smoking and drinking histories between subjects and their controls, however, was likely comparable because both groups were outpatients of comparable age receiving care at medical institutions for a disease (hospital‐based case–control study). Any misclassification of smoking and drinking histories caused by improper recall would likely have balanced out between subjects and their controls (i.e., amounting to nondifferential misclassification) and thus would not have affected the results. Second, controls were selected from the outpatient services of tertiary medical institutions and may have had characteristics differing from those of the general population. This issue was addressed by excluding certain diseases that could have affected the results (malignancies, Crohn's disease) and including patients with a wide range of conditions in addition to patients with a gastroenterologic disease as controls. Additionally, the proportion of current smokers in the control group was 29% for males and 16% for females, which was not considered to differ significantly from distributions in the general population (adult smoking rates in Japan from 2008 to 2013 were 32–38% for men and 8–11% for women^26^). Third, we may not have been able to detect relevant factors due to the small sample size. Sample size calculations as a post hoc analysis showed that a total of 281 subjects (94 cases and 187 controls) were required to detect a significant association between ex‐smoking and UC development with an α level of 0.05 and a β level of 0.20. To detect any significant association between ex‐drinking and UC development, a total of 440 subjects (147 cases and 293 controls) were required. Therefore, the number of subjects may have been small to investigate the association between ex‐drinking and UC development. Finally, lifestyles such as smoking and drinking habits are thought to be strongly influenced by socioeconomic factors, but such factors could not be included in the model as adjusted factors in this study.

## Conclusions

The present multicenter case–control study using incident (new) cases of UC showed a higher OR for UC among ex‐smokers and ex‐drinkers. Our findings suggest the need for careful attention for UC onset in heavy smokers who quit smoking before 40 years of age and in heavy drinkers who quit drinking at or after 40 years.
